# Perceptions of LGBQ+ youth and experts of suicide prevention video messages targeting LGBQ+ youth: qualitative study

**DOI:** 10.1186/s12889-020-09853-5

**Published:** 2020-12-02

**Authors:** Stefanie Kirchner, Benedikt Till, Martin Plöderl, Thomas Niederkrotenthaler

**Affiliations:** 1 Department for Social and Preventive Medicine, Medical University of Vienna, Center for Public Health, Unit Suicide Research & Mental Health Promotion, Kinderspitalgasse 15/1, 1090 Vienna, Austria; 2 Department for Crisis Intervention and Suicide Prevention, Christian Doppler Clinic, Paracelsus Medical University, Ignaz-Harrer-Straße 79, 5020 Salzburg, Austria

**Keywords:** LGBTQ+, Youth, Suicide, Suicide prevention videos, Focus groups, It gets better, Media, Suicide prevention, Perception, Qualitative study

## Abstract

**Background:**

Young lesbian, gay, bisexual, transgender, queer as well as other sexual/gender minorities (LGBTQ+) persons have higher rates of suicidal ideation and behavior compared to their non-LGBTQ+ peers, particularly during their coming out. The “It Gets Better” project is a multi-national media campaign that aims to reduce suicide among LGBTQ+ adolescents by providing personal narratives of hope delivered by mainly adult LGBTQ+ persons. There is only little knowledge so far on how young LGBTQ+ people as well as experts in suicide prevention and counseling perceive these videos, and how to potentially improve the videos based on their perceptions.

**Methods:**

A total of *n =* 19 LGBQ+ adolescents and young adults and *n =* 9 experts participated in focus groups to discuss perceptions of a selection of “It Gets Better” videos. Eight focus groups were conducted to assess perceptions on the process of watching the videos, possible effects on young LGBQ+ viewers in general, and suicidal LGBQ+ youth in particular, as well as factors that were relevant to their perceptions.

**Results:**

Messages were found to be helpful in terms of promoting hope. LGBQ+ youth identified several key strategies to increase identification with messages, which they considered crucial for their effectiveness. Criticism emerged from a perceived lack of diversity in terms of portrayed sexual identities, and some shallowness in the portrayal of suicidal ideation and how things can get better. The experts’ perceptions of the videos were largely consistent with LGBQ+ youth, highlighting a positive potential of videos to support coming out and identity building processes.

**Conclusions:**

Young people and experts view the videos as helpful and relevant, but identified several strategies to better tailor them to the needs of LGBTQ+ adolescents, including suicidal peers. The insights gained are useful to the increasing number of suicide prevention projects using personal narratives of coping delivered via media to help prevent suicide.

**Supplementary Information:**

**Supplementary information** accompanies this paper at 10.1186/s12889-020-09853-5.

## Background

Adolescents from sexual minorities, including gay, lesbian, bisexual, transgender, queer as well as other sexual/gender minorities (LGBTQ+) have a higher vulnerability for suicidal ideation, suicide attempts, and suicide than their non-LGBTQ+ peers [1, 2]. The risk has been found to be particularly pronounced around the time of coming out [3]. Various stressors such as rejection, discrimination, or negative attitudes towards one’s own identity shape this period in life [4, 5].

Acceptance by family, friends and others and having a strong affiliation to the LGBTQ+ community are important aspects in facilitating the process of coming out and developing one’s own sexual identity [6, 7]. Access to these resources is sometimes limited during adolescence and many LGBTQ+ youth still fear rejection from their peers or family [8]. In this context, media and digital communication are important [9], because they can help identifying peers and positive role models who might not be readily available in the physically close environment [10–12]. Online media therefore often represent a suitable means to explore one’s identity, find like-minded people, communicate with others, share personal experiences, and access resources, thus facilitating identity development [13, 14].

Personal narratives of hope and recovery in media have also been found to be a potentially useful tool in suicide prevention [15]. Importantly, such narratives might reduce suicidal ideation in the audience specifically by increasing coping beliefs, a phenomenon that is called “Papageno effect” [16–21].

Although evaluations of media campaigns targeting LGBTQ+ individuals are very scarce [9, 22], there are reasons to hypothesize that they might be particularly useful for adolescents in exploring and building their sexual identities. Engaging online and viewing lived experiences of others who managed to cope with adversities has been shown to positively affect viewers’ offline lives and presents a substantial part in the process of coming out [14]. Narratives of how to cope with adverse circumstances and connect with the community might thereby increase resilience to adverse experiences during identity-building [23].

The “It Gets Better” project (IGBP) was one of the first media campaigns specifically targeting LGBTQ+ youth [24]. It started in 2010 in the United States after several suicides by young people who were bullied for being gay. Its core aim is to provide hope and prevent suicide among affected adolescents [25]. The IGBP features personal narratives of mainly adult LGBTQ+ individuals who describe how they managed to cope with adversities experienced during their adolescence and coming out. The project constitutes a collective community action campaign calling everyone to participate and contribute [26]. This way it is conceptualized as a form of community for young LGBTQ+ adolescents facing minority-related adversities [9]. The project was adopted in several countries. Austria was the first and only German language European country so far to implement the IGBP in 2013 [27].

In terms of video contents in the IGBP, there is one study available that analyzed the contents of German-language videos [28], and several studies on American IGBP videos [9, 23, 29–38]. The Austrian IGBP was found to mainly focus on personal narratives related to coming out and instilling hope. Self-acceptance and having a supportive environment are typically highlighted as crucial factors for making life better in the videos, whereas questions on how to resolve a suicidal crisis as well as resources for getting professional help were only rarely addressed [28].

Up until now, there is very little knowledge on how LGBTQ+ individuals as well as experts experience and perceive the IGBP videos. Only few studies qualitatively examined the IGBP from the viewpoint of LGBTQ+ adolescents, and these analyses are only available for videos from the American IGBP [23]. They suggested that the project increased awareness of the problems LGBTQ+ youth have to face, but also noted a lack of attention to issues of socio-cultural diversity [30].

The present study is the first study to assess the individual perceptions of LGBQ+ youth and experts of selected ideal-typical videos from the German-language IGBP. This is also the first study worldwide to address both perspectives from LGBQ+ youth and experts in the areas of suicide prevention and counseling of LGBTQ+ youth.

In our study we addressed the following research questions:

1. How did participants feel while watching and rating the videos?
2. What was important to the participants while rating the videos?
3. What are the participants’ thoughts on enhancing any positive effects?
4. What are specific suggestions regarding the videos’ usefulness for suicide prevention?

## Method

As described in a previous analysis [28], videos in the IGBP Austria were heterogeneous, ranging from short inputs of a few seconds with calls to “hang in there” to detailed personal narratives of how to cope with adversities. Based on potential beneficial effects of media messages covering ways how to cope with suicidal ideation [39], we defined the following criteria beforehand to do a first pre-selection of videos for the focus groups: (i) the video features at least one LGBQ+ person; (ii) the focus is on a personal narrative; (iii) the focus is on difficulties during adolescence and/or during coming-out; and (iv) emphasis is put on how things got better. All *n =* 198 videos from the Austrian “It Gets Better” project were screened for the defined criteria by the first author and independently by the senior author. Afterwards, the pre-selected videos (*n =* 20) were shared with the research team and critically discussed. Videos that contained vague descriptions of how life got better and videos that did not reach full consensus regarding their fulfillment of all inclusion criteria were subsequently excluded. Based on the discussion, a total of *n =* 7 videos with *n =* 4 videos featuring male protagonists and *n =* 3 female protagonists were considered to fully meet the inclusion criteria. In the next step, a jury of *n =* 28 participants including *n* = 19 LGBQ+ youth and *n* = 9 experts was recruited and invited to select the most suitable videos from the pre-selected 7 videos. The rating was done separately for videos with male and female protagonists, i.e. female jury members rated videos featuring female protagonists and male participants rated videos featuring male protagonists. To investigate the research questions, all participating individuals were invited to participate in focus groups immediately after the rating.

### Participants

We recruited LGBQ+ youth at two branches (Vienna, Salzburg) of the “Homosexuellen-Initiative” (HOSI), Austria’s major LGBTQ+ organization who also have youth-groups, between February and April 2019. We included all adolescents/young adults aged between 14 and 27 who were identifying as LGBQ+ (lesbian, gay, bisexual, queer or questioning or from other sexual minorities), and who were not currently experiencing severe suicidal ideation. In this study, we only included cisgender youth as the selected videos did not cover transgender or nonbinary individuals, and identification with the featured protagonists was therefore assumed to be stronger for cisgender youth. The inclusion and exclusion criteria as well as suicidality or current suicidal ideation were assessed in a structured interview with the first author at first contact with the participants. The participants were informed about the aim of the study and its focus on suicide prevention. Interested individuals were asked if they had experienced suicidal ideation in the past or if they currently experienced suicidal ideation. The first author discussed the study requirements and exposure to questions on suicide with all participants before making a final decision on participation with the participant. None of the individuals who wanted to participate indicated current suicidal ideation, and no participant was excluded. Austrian experts with experience in suicide prevention media campaigns, LGBTQ+ health research, and/or counseling of LGBTQ+ individuals were also invited from the pool of members of the expert committee currently implementing the Austrian suicide prevention plan, as well as from LGBTQ+ specific counseling organizations.

After obtaining written informed consent, a total of *n =* 19 adolescents/young adults (male: *n =* 17; 60.7%; female: *n =* 11; 39.3%) and *n =* 9 experts (male: *n =* 5; 55.6%; female: *n =* 4; 44.4%) agreed to participate in the focus groups. The median age of the LGBQ+ youth was 22 years (*IQR =* 5). The adolescents/young adults identified mainly as gay (*n =* 10; 35.7%). Two identified as bisexual (10.5%), two as queer (10.5%) and one as pansexual (5.3%). No information was available for four adolescents/young adults (21.1%). Two of the participants (10.5%) reported past suicidal ideation.

The group of experts was selected based on their professional experience in suicide prevention or LGBTQ+ counseling. The group specifically included experts from the field of media and mental health promotion/suicide prevention (*n =* 4); gender studies (*n =* 1); and also included clinical psychologists working with LGBTQ+ adolescents (*n =* 2) or suicidal adults (*n =* 2).

### Procedure/focus groups

In order to prepare LGBQ+ youth to the task of discussing the videos regarding their appropriateness for suicide prevention, a short introduction session before the rating process and focus groups was deemed necessary. This was kept very brief in order not to unnecessarily influence participants. The participants were educated on selected basics of suicide prevention and informed about the procedure of their study participation and the rating of the videos. The introduction session included the addressing of the most common public suicide myths (e.g., if someone is suicidal, they are suicidal forever; if someone speaks about suicide, he/she is at lowest risk) and informing them about basics about what is currently known about media effects on suicide from non-LGBTQ+ specific settings.

All participants were then provided with an online link containing the videos and a related questionnaire and were asked to complete this before the focus group discussions. The questionnaire covered aspects on the suitability of the videos to reduce suicidality, increase help-seeking behavior, strengthen one’s sexual identity, and increase hope [see Additional file 1]. The participants were asked to rate the videos regarding these pre-defined criteria in order to identify the best videos.

The subsequent discussions focused on four main topics:

- own experiences while watching the videos;
- perceptions about effects on other LGBTQ+ adolescents during coming out;
- what factors were deemed relevant in their perceptions; and
- perceptions about possible effects on suicidal LGBTQ+ adolescents

A total of 8 focus groups were conducted by the first author between March and April 2019. The first author is experienced in the conduction of semi-structured interviews and received additional training prior to the conduction of the focus groups. The focus groups, which lasted between 51 and 98 min, were conducted separately for adolescents and for experts in order to prevent experts exerting undue influence on youth. We also separated male and female participants, who discussed videos featuring either male (*n =* 4 videos) or female (*n =* 3 videos) protagonists, to account for the possibility that identification with the featured protagonist might vary with the gender of the portrayed individuals. Each focus group comprised between two and five participants, with most of them including 4 participants (*MD =* 3.5; *IQR =* 3). The small number of participants per group enabled us to gain more in-depth insight into the participants’ perceptions and opinions as compared to larger groups [40]. The groups were instructed that an open discussion and sharing of experiences/views with other group members were welcomed. A semi-structured interview guide, which was developed by the research team, was used only when necessary (e.g., if the discussion came to a halt; see Additional file 1). The focus groups were audio recorded with a digital recorder.

### Analysis

The focus groups were analyzed using the documentary method [41]. This method takes the participants’ experiences, both in terms of content of the conversation and the form in which it is presented into account [42].

The materials were fully transcribed and read in depth by the first and the senior authors, who identified major codes as they related to the research questions. Subsequently, subcodes were assigned to each statement, i.e. each full statement raised in the discussion was a unit of analysis. Subcodes served to summarize specific aspects of a code, e.g. related to “identification” as one of the factors that were deemed relevant to individual perceptions of the videos. For some topics, where more detailed in-depth discussion occurred, further levels of detail were coded. In some instances it was also deemed helpful to define code families based on cross-connections between codes as they came up in the discussions. Any discrepancies between the first and senior authors were discussed and resolved.

In order to assess saturation of the material [43], the first and senior authors did a post-hoc evaluation and found data to be saturated after five (of eight) focus groups, meaning that all statements from focus group number 6 onwards were readily assignable to existing codes without generating further new codes. Data saturation after the fifth focus group is consistent with what has been found in other qualitative research [44].

## Results

### Group dynamics and discourse

The group dynamics were harmonious and participants were engaged in the discussion with other group members. The participants generally agreed to other group members’ statements and explicit dissent was rare. Thematic shifts were mostly initiated by the interviewer in case no new aspects were brought up.

### Adolescents

#### Experiences while watching the videos

**Fig. 1:**
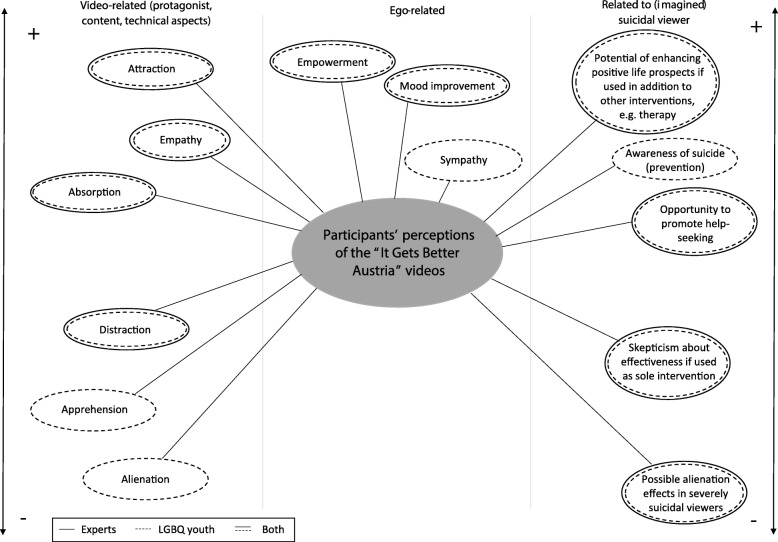
Participants’ perceptions of the “It Gets Better Austria” videos. The individuals’ perceptions were categorized into perceptions related to the video (e.g., technical aspects, protagonist, content), perceptions more related to themselves, as well as perceptions related to effects on (imagined) suicidal viewers. The perceptions were organized on a scale from positive to negative based on their connotations. Both LGBQ+ youth and experts’ perceptions are shown in the figure, and, where applicable, consent between the two groups is indicated. Categories as shown in the figure are written in italics and marked with “->” in the main body of the manuscript for easy identification of related text

In general, participants reported having been - > *absorbed* (Fig. 1) by the videos and engaged with the content. While watching the videos adolescents/young adults reported an - > *improved mood* and feelings of - > *empowerment* (see Fig. 1).

Many participants reported that they felt - > *empathy* for the protagonists and tried to put themselves in the shoes of LGBTQ+ adolescents who were currently in their coming out and may experience distress. Some participants went a step further and were strongly reminded of their own coming out and own past situations and feelings, which triggered - > *sympathy* for the protagonists or their narratives and resulted in a memory of own past situations and feelings related to their own coming out.

*” I somehow felt a little bit like I was propelled back to my own coming out, because you start thinking, okay, how was my own coming out, how was my process, […*.*]* *and it was actually interesting.”* [Male, age 27, gay].

The participants expressed that they felt - > *attracted* to the videos. Specifically, they appreciated the sharing of authentic personal narratives. Many adolescents/young adults experienced feelings of hope and not being alone.

“*… Somehow it triggered a feeling of solidarity, because I could identify with different stories … [ …]*. *Indeed I felt a little bit less lonely and I thought, okay, it’s actually normal what I feel or how I feel …*” [Male, age 22, gay].

Some aspects, on the other hand, were perceived as scary and daunting, resulting in some sort of - > *apprehension*. Some participants specifically argued that aspects of the viewed content, particularly difficult life situations that were portrayed, might lead to viewers distancing themselves from the videos or leaving them discouraged.

*“Like this [narrative], I was bullied for years [ …] and then I think, well, if I listen to this now, I would think twice if I rather not just keep this [sexual orientation] to myself, if I will be bullied at school afterwards, so when I think, okay, I already feel bad, and then I probably will be bullied, then I think this is a little bit too much.”* [Female, age 24, bisexual].

**Fig. 2:**
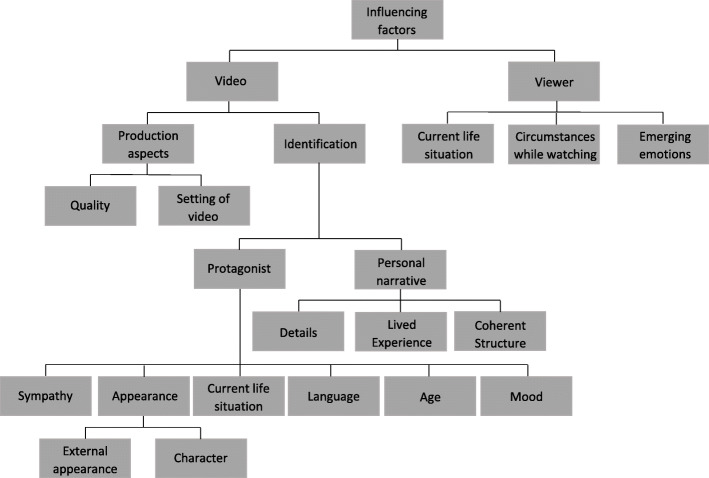
Factors identified by participants to be influencing their perceptions of the videos. Influencing factors were categorized into code families either related to the viewer (his or her current life situation, circumstances while watching, and emotional state) or to the video (production aspects and factors relevant to identification with the specific narrative). With regard to production aspects, the quality of videos and the setting were considered important. With regard to factors relevant for identification, aspects of the featured protagonist and the specific personal narrative were deemed relevant. In particular, the narrative was better perceived if it was structured in a coherent and comprehensible way, and if it covered details of lived experience. Sympathy for the protagonist, his or her external appearance and perceptions of his or her character, resonance with the current life situation as portrayed in the video, his or her language and age as well as a positive mood were further relevant factors for identification

A strong focus in the discussions was put on how to increase any positive effects of the videos on young viewers. The different code families, codes, subcodes and further subcategorizations of factors deemed relevant to increase a positive effect are visualized in Fig. 2. These included factors specific to the video and factors more related to the viewer. The viewer was regarded as important in terms of the current life situation, particularly their stage of coming out at time of viewing, their specific life circumstances and their emotional state.

*“There are probably no objective criteria, so surely this is dependent on the type of person who’s watching and simply on the current situation or so, so I think there’s no standard recipe, like, which is 100% right for everybody.”* [Male, age 25, gay].

The most important influencing factor repeatedly emphasized was the necessity to offer opportunities to identify with the featured characters. The participants stated that they were able to either identify with the protagonist himself/herself or his or her personal narrative. External appearance, likeability as well as the current life situation of the protagonist such as whether his or her life situation resonated with the viewers’ own life situation or values were deemed relevant. Having the same sexual or gender identity was also identified as a crucial aspect in the identification process. Participants noted that specific sexual or gender identities were linked to specific challenges and the portrayal of this diversity was essential.

*“It’s really good that there were different scenarios and that, because if you give general advice about coming out, then it’s … you can’t really go into one scenario, that’s relative and it’s different for everybody, but you may familiarize with it, does that apply to me, are people from my social environment similar, may I expect a similar outcome, and I think it’s great that the adolescents have comparisons.”* [Female, age 17, lesbian/queer].

Demographic aspects such as the protagonist’s age and language were also discussed as important for identification. The participants perceived narratives of older protagonists not to be fully applicable to life today and not generally helpful for LGBTQ+ adolescents.

*“In this video it was principally difficult to identify with him, because of a different target group or simply [because he’s] older, and he grew up in a different time. So, I don’t want to imply that it was easier back then compared to nowadays or maybe not equal, that’s a different situation [ …] but I couldn’t really identify with this video”* [Male, age 22, gay].

On the level of the personal narrative, the participants noted that details were essential and criticized statements they perceived as too general or shallow. Some adolescents/young adults described a feeling of annoyance when confronted with superficial encouragements, thus leading to a feeling of - > *alienation* (see Fig. 1). Most participants emphasized the importance of depictions of lived experiences by first outlining past adverse circumstances followed by a detailed description of how life gets better.

*“Well, I think, at least for me it’s very important, that at first a connection is established. [ …] That they also had concerns or fears, worries what might happen, what … how will people react. And I think, this is the most important start to simply show you were in the same situation. [ …] Starting from this, you talk about your experiences and … and you show how everything developed. And it doesn’t always have to be positive. So especially for negative things, I think it’s important that people tell how they coped with it and what they did, to better cope with it. That’s particularly interesting.”* [Male, age 19, gay].

Participants repeatedly criticized how non-gay and non-lesbian sexual and gender identities were displayed in the videos. Specifically, one protagonist in a video portrayed bisexuality as a transition to becoming gay/lesbian. Adolescents/young adults pointed out that the different characteristics of the LGBTQ+ groups should be considered in the production of the videos and had to be more carefully addressed.

*“There was one guy, where it really bothered me, the way he presented bisexuality, because virtually it’s somehow, um, so it’s virtually not an option but rather, you are either gay or simply lesbian, or simply hetero, and bisexuality is virtually only a transition or so, that’s also a wrong message, because it’s not true. I mean, there are many, who do not come out immediately, who don’t dare to come out as gay, and, um, that’s clear, but there are very well people who are in fact bi, and they should also feel addressed, I think.”* [Male, age 22, gay].

The adolescents/young adults felt strong - > *empathy* for the protagonists or their narrative. In particular, a “happy ending” was perceived as very important by participants, and videos sparked a sense of sadness in some participants if they did not perceive the video to have a happy ending. The participants concluded that personal narratives should be positive but at the same time not diminish negative aspects in life. Some videos were found to be somewhat too negative by portraying a situation that would not necessarily apply to each viewer and might intimidate some viewers (e.g., a negative coming out, being bullied, and having no other choice but to find new friends).

Production aspects of the videos were also frequently discussed. Many participants complained about low production quality, emphasizing that it reduced the impact of the respective narrative as they felt - > *distracted* (Fig. 1) and thus could not pay full attention to the content.

#### Suitability for Suicide Prevention: Effects on (imagined) suicidal peers

Most participants perceived the addressing of suicidality as crucial and rated it positive if present in the video while simultaneously criticizing the weak focus on suicidality in the selected videos.*“So, if I hadn’t known that this was about prevention, especially for, I don’t know, like, suicidal ideation or so, I wouldn’t have noticed. So, they kind of talked about it, but it was so short that it wasn’t really focus. So, if you don’t know, what it’s all about, no idea.”* [Male, age 22, gay].

Participants also emphasized the importance of better addressing professional help services in order to make the videos useful specifically for suicidal peers.

*“So, it wouldn’t have been bad to include this [professional resources] maybe, like, okay, now I have suicidal ideation, I don’t know, I feel bad. The video helped me somewhat, but what do I do now? Now I feel a little bit better, now I may be motivated to confide to someone or so or go somewhere. But if I don’t know where that is, it will be difficult.” [Male, age 27, gay].*

Possible benefits for suicidal peers in terms of enhancing - > *positive life prospects* by watching the videos were considered particularly relevant if videos were used as a first step to seek help or as complementary to some type of professional support. The potential usefulness of videos to provide a low-threshold *- > opportunity to promote help-seeking* was frequently noted. On the other hand, participants expressed some *- > skepticism* about the potential of videos to reduce suicidal ideation particularly if they were used as the sole intervention to address suicidality. Further, the videos were found to give a positive life prospect and a feeling of manageability. Some adolescents/young adults also identified the videos to be potentially suitable for increasing - > *awareness* of the social environment and situation of peers affected by suicidal ideation.

*“I think the videos are also very important … not only for those affected [by suicidal ideation] themselves, but also for the social environment of the affected, because then they finally see how straining it can be, when you are not accepted for your sexuality. Yes, so, that there’s a lot of pressure and that people, who are simply not queer, can’t understand.”* [Female, age 16, pansexual/no label].

With regard to the suitability of specific narratives used, an important factor to influence any effect of the videos on suicidal peers was deemed to be the viewer’s degree of suicidality. Some adolescents/young adults were concerned about possible adverse effects in severely suicidal individuals, particularly - > *alienation* effects. Specifically, adolescents/young adults noted that suicidal peers might feel alienated by a portrayal of someone living a good life, because this situation might appear unattainable for him or her at this point of their lives.

*“I think if I were about to attempt suicide and I would have watched the second video [a video featuring a happy couple], then I would have felt even worse afterwards, because then I would think, okay, they are feeling great, there never happened anything bad to them, so why can’t I have that?”* [Female, age 16, pansexual/no label].

### Experts

#### Experiences while watching the videos

In general, the experts addressed similar aspects with regards to their perceptions of the videos (see Fig. 1). Most of the experts noted that the videos might have a strong positive potential on LGBTQ+ adolescents with regard to assisting them in their coming out and identity building.

Consistent with statements of adolescents/youth, experts also felt strongly about the potential of videos to increase identification with the featured protagonists, and the helpful potential of such identification processes.

Regarding putatively effective personal narratives, expert participants emphasized the need of a “low point” in the narrative in order to link to LGBTQ+ adolescents facing adversities. Experts noted that they felt identification of adolescents with the respective protagonists was unlikely if adversities were not addressed in the video or if difficulties were shared in a way that portrayed them as being too far in the past, creating distance between the protagonist and the audience.

#### Suitability for suicide prevention: effects on (imagined) suicidal peers

The range of difficulties portrayed solely focused on coming out, which was critically reflected on in the expert discussions and a source of *- > skepticism* regarding their effectiveness. Further, the campaign’s focus on coming out as (seemingly) sole LGBTQ+ related difficulty was criticized. Coming out was discussed not to be the only factor in suicidal ideation, and the need to include other LGBTQ+ (and even non-LGBTQ+ specific) relevant issues beyond coming out, such as e.g. mobbing or alienation from peers, or mental illness, was repeatedly noted.

Many experts also addressed the need to explicitly talk about suicidal ideation to tailor the videos to suicide prevention and pointed to the multitude of opportunities for a positive framing of videos, which would still allow to address suicidality more explicitly.

*“[ …] and perhaps using the example [of one of the protagonists in the video] you can see that there’s a misunderstanding that suicidality does not need to be negatively framed … maybe it’s an ad hoc understanding and probably very commonly associated, however, you can indeed frame suicidality positively by showing: that’s how I felt and then … that’s how I got out of it …”* [Male, researcher].

Based on the findings of the focus group discussions, the following recommendations were derived from the discussions on how the videos could be improved for addressing suicide prevention (see Table 1).

**Table 1:** Recommendations on how to improve the videos brought up by the participants

General Aspects of the Video	
• Feature various protagonists of different LGBTQ+ groups and gender identities as well as different personal styles and appearances to offer opportunities for identification	
• Consider different characteristics of LGBTQ+ groups in the production of the videos and address their specific issues carefully (e.g., specific issues bisexual adolescents have to face)	
• Authentic appearance and narrative of the protagonist	
• Videos should have a certain level of quality but not be overproduced (no background noise or blurry pictures)	
Narrative	
• Feature different narratives to offer opportunities for identification	
• Feature a lived experience: provide a narrative that starts by outlining briefly past adverse circumstances (a “low point”) followed by a detailed description of how life got or is now better	
• Details are essential, and advice or ways on how to overcome a crisis should be specific	
• Personal narrative should be positive, but should not diminish negative aspects in life; videos should have a “happy end”	
• Address issues typically encountered during adolescence which are not necessarily only LGBTQ+ related	
Suicidality	
• Address suicidality explicitly using a positive framing: describe a suicidal crisis and show ways how to positively cope with it	
• Provide resources to professional help services in the videos in order to assist with suicidal ideation and behaviors	

## Discussion

The present article revealed important insight into how LGBQ+ individuals experience German language “It Gets Better” videos. The videos were experienced and judged to promote feelings of hope and not being alone as a young LGBTQ+ person. LGBQ+ youth felt attracted by the videos, felt empathy or sympathy for the protagonists, experienced improvements in mood, and felt absorbed by the videos. On a more negative side, some adolescents and young adults felt distracted by specific characteristics of videos, mainly technical aspects, or felt some sort of apprehension related to scary contents and alienation. The weak focus on suicidality and professional help-seeking was frequently identified as an area for improvement. Several aspects were identified that might help in creating effective videos, which were closely related to the question how videos can generate identification with the featured characters or their featured narrative. Relevant aspects included behavioral and emotional characteristics of the viewers (e.g., his/her current life situation and circumstances) as well as factors related to the video itself (video setting, quality).

The Austrian “It Gets Better” project (IGBP) was identified to have potential to raise awareness regarding the life situation of LGBTQ+ youth. This finding is consistent with an analysis of the perceptions of American LGBTQ+ adolescents of the US IGBP [23]. Also consistent with findings of the American IGBP [23], the Austrian IGBP was not always recognized as a suicide prevention project by LGBQ+ youth. This finding is concerning and highlights the need to better address suicidal ideation and suicide prevention in the videos.

LGBQ+ adolescents/young adults were afraid that some overly negative situations portrayed in the videos might instill anxiety in some peers during or before their coming out if they were focused on very unique and troublesome life situations. Both LGBQ+ youth and experts feared negative effects if depicted situations appeared very negative [30]. On the other hand, participants also emphasized that narratives should not be shallow or too positive in order to enhance identification with the narrative.

Interestingly, in research on the American IGBP, concerns were primarily about portrayals being potentially too positive. Specifically, the positive stories were discussed to potentially lead some viewers to take too many risks for their coming out, which might possibly result in poor outcomes [23]. These and our present findings suggest a fine line of portraying LGBTQ+ youth’s problems resulting in a need to find a balance between positive and negative depictions in a video message.

Both LGBQ+ youth and experts criticized the Austrian IGBP to be too narrowly focused on coming out-related issues (e.g. how to come out to your family and friends, fear of being rejected), while not sufficiently addressing other common challenges for LGBTQ+ youth and suicidal youth. This was different from findings for the American IGBP, which was perceived to create awareness particularly for suicide and bullying [23]. Differences in the predominant narratives in the two IGBP campaigns likely explain these findings. American IGBP video messages typically focused on adversities such as bullying, whereas the Austrian IGBP was focused on coming out stories linked with hopeful messages [28].

The need for more diversity by featuring more non-gay and non-lesbian sexual and gender identities and their specific issues which was highlighted in the present focus groups is consistent with a study by Craig et al. [23] in the United States. This study highlighted a similar lack of depicting LGBTQ+ identities beyond gay and lesbian characters, particularly of transgender people.

The participants identified many factors which might influence the effects of the videos, and most of them were related to their potential of resulting in identification with the featured protagonists. These findings are consistent with studies by Bandura [45] and Cohen [46], who found identification to be enhanced by similarities between featured protagonists and viewers. A study by Slater and Rouner [47] found that the extent to which a viewer engages with the narrative was important and varied with his or her personal interest and involvement with the aspects portrayed. Thus, a narrative not directly relevant to the viewer is unlikely to receive full attention [48].

In general, both LGBQ+ adolescents/young adults and experts noted potentially positive effects of portrayals of overcoming a crisis situation, and the scarcity of such positive framing of suicidal ideation and suicide prevention in the videos was a main point of criticism. Watching others succeed and mastering their crises might give the viewers confidence and an incentive to take action themselves, e.g. in terms of seeking professional help if suicidal [49]. Providing specific coping strategies has been previously discussed to increase the power of the message and make prevention videos more effective [48]. Both LGBQ+ youth and experts frequently recommended to portray personal lived experiences of how to master a suicidal crisis, and this approach has also been suggested as *the way forward* in recommendations for suicide prevention [50]. Recent research suggesting beneficial potentials of stories featuring ways to overcome suicidal ideation, the “Papageno effect”, further emphasize this strategy [16–18].

### Strengths and limitations

A strength of the study is that both LGBQ+ youth as well as experts were included in the study and perspectives from both groups were captured. Themes that were identified by youth were largely consistent with experts.

However, the study also had some limitations. The median age of the LGBQ+ participants was 20 and only two participants were minors. This age span covers different developmental phases, which might have a strong impact in perceptions of the videos. Most participants had their coming out several years ago and were well-connected to the LGBTQ+ community. Their perceptions could be different to adolescents who may not be (fully) connected to the community or who might not know any other LGBTQ+ peer personally.

While the American IGBP faced a lot of criticism as highlighted in a previous study [23], no major points of criticism were brought up in the present focus groups. This might indicate specifics of the selected individuals, differences between the Austrian and US-American IGBP, or a reluctance to criticize the IGBP due to group dynamics. Explicit dissent was rare and some opinions might have been underrepresented in the focus groups.

Another limitation was that the discussed videos were not representative of the total pool of the Austrian IGBP videos. The findings therefore apply to videos that adopted the preselection criteria, but not necessarily to all IGBP videos.

A further study limitation was that, in instances of no explicit disclosure of gender identities in the respective video (e.g., the featured protagonists identify as male, female or other), the research team was not able to make firm conclusions about the gender identities of the featured individuals.

Finally, important basic aspects on the current evidence of media impacts on suicide were shared with the young participants before the rating task to achieve maximum benefits in terms of the study aim, i.e. to identify areas of improvement for videos for suicide prevention. Some adolescent/young adult views might have been different if they had not been exposed to the introduction beforehand.

## Conclusion

The Austrian IGBP resulted in many positive perceptions among LGBQ+ youth and prevention experts. According to LGBQ+ youth and experts, portrayals of lived experiences with adversities during coming out should provide a wide range of opportunities for identification to resonate with LGBQ+ adolescents struggling with their identity building and their coming out. Suggestions for main areas for improvement include a stronger focus on suicidal ideation and ways to cope with it. A wider variety of protagonists and narratives, both in terms of sexual identities and orientations, as well as individual life stories, is needed to broaden the spectrum of the campaign. The findings of this study, which is the first to address both perceptions of LGBQ+ youth and prevention experts, are of immediate relevance to any suicide prevention project targeting sexual minorities and have broader implications for any suicide prevention project featuring individual stories of hope and recovery.

## Supplementary information

Additional file 1 This file entails all materials developed for this study and used to collect the data.

## Data Availability

The materials developed for this study are provided in the additional material 1. The data that support the findings of this study are available on reasonable request from the corresponding author [T.N.]. The transcripts are not publicly available due to their containing information that could compromise the privacy of research participants.

## Abbreviations

LGBQ+: Lesbian, gay, bisexual, queer as well as other sexual/gender minorities
LGBTQ+: Lesbian, gay, bisexual, transgender, queer as well as other sexual/gender minorities
IGBP: “It Gets Better” project
