# Supplementary material for: Perceptions of LGBQ+ youth and experts of suicide prevention video messages targeting LGBQ+ youth: qualitative study
Source: BMC Public Health. 2020 Dec 2;20:1845. doi: 10.1186/s12889-020-09853-5 (PMC7709299; doi:10.1186/s12889-020-09853-5)
Supplement: Supplementary file 1 — This file entails all materials developed for this study and used to collect the data. [file 12889_2020_9853_MOESM1_ESM.docx]

Additional File 1

Table of Contents

Online Questionnaire p. 2

Interview Guide Focus Groups p. 6

Links of the videos that were discussed in the focus groups p. 8

Online Questionnaire

Dear participant,

Thank you very much for willing to participate as jury member in our study “Effects of suicide prevention videos targeting LGBQ adolescents”.

Please take some time to watch the videos on the following webpages.

These videos were pre-selected based on following criteria:

- The person featured in this video is either gay, lesbian or bisexual.
- The person featured in this video shares a personal narrative.
- The content of the video is about adversities encountered during one´s adolescence and/or during one´s coming out.
- The video contains a description of how life got better for the featured protagonist.

On the following webpages you will find a small questionnaire for each video. These questions should assist you in your rating. We recommend to fill out each questionnaire directly after watching the respective video.

Afterwards the most suitable video will be shown to LGBQ adolescents who are in the course of coming out. Hence, please try to empathize with them and ask yourself if this video would be suitable for these adolescents. Do you know a person in your social environment, who is in a similar situation?

Please fill out this questionnaire prior to the start of the focus group, as the results will influence its structure.

Video 1

1. **How did you like the video in general?**

| ⃝  Not at all | ⃝  Not really | ⃝  Neither … nor | ⃝  Somewhat | ⃝  Very much |
| --- | --- | --- | --- | --- |

1. **How comprehensible was the video for you?**

| ⃝  Not comprehensible at all | ⃝  Not comprehensible | ⃝  Neither … nor | ⃝  Comprehensible | ⃝  Very comprehensible |
| --- | --- | --- | --- | --- |

1. **How coherent was the narrative for you?**

| ⃝  Not coherent at all | ⃝  Not coherent | ⃝  Neither … nor | ⃝  Coherent | ⃝  Very coherent |
| --- | --- | --- | --- | --- |

1. **How much could you identify with the featured protagonist?**

| ⃝  Not at all | ⃝  Not really | ⃝  Neither … nor | ⃝  Somewhat | ⃝  Very much |
| --- | --- | --- | --- | --- |

1. **How would you rate the quality of the video?**

| ⃝  Not valuable at all | ⃝  Not valuable | ⃝  Neither … nor | ⃝  Valuable | ⃝  Very valuable |
| --- | --- | --- | --- | --- |

1. **In your opinion, how suitable is this video for LGBQ adolescents in their coming-out to …**

|  | Not suitable at all | Not suitable | Neither … nor | Suitable | Very suitable |
| --- | --- | --- | --- | --- | --- |
| Reduce suicidal ideation | ⃝ | ⃝ | ⃝ | ⃝ | ⃝ |
| Facilitate help-seeking | ⃝ | ⃝ | ⃝ | ⃝ | ⃝ |
| Reduce negative attitudes towards oneself | ⃝ | ⃝ | ⃝ | ⃝ | ⃝ |
| Facilitate acceptance towards one´s own sexual identity | ⃝ | ⃝ | ⃝ | ⃝ | ⃝ |

1. **In your opinion, how suitable is this video to provide hope to an LGBQ adolescent who is currently in a difficult situation?**

| ⃝  Not suitable at all | ⃝  Not suitable | ⃝  Neither … nor | ⃝  Suitable | ⃝  Very suitable |
| --- | --- | --- | --- | --- |

1. **In your opinion, how suitable is this video for other Austrian LGBQ adolescents, who encounter difficulties during their coming out, to identify with the featured protagonist?**

| ⃝  Not suitable at all | ⃝  Not suitable | ⃝  Neither … nor | ⃝  Suitable | ⃝  Very suitable |
| --- | --- | --- | --- | --- |

1. **In your opinion which group within the LGBQ community would benefit most from this video?**

______________________________________________________________________________________________________________________________________________________________

1. **Was there something in this video which you particularly liked?**

⃝ Yes ⃝ No

If so, what was it?

______________________________________________________________________________________________________________________________________________________________

1. **Was there something in this video which particularly bothered you?**

⃝ Yes ⃝ No

If so, what was it?

______________________________________________________________________________________________________________________________________________________________

[Questions 1. to 11. are repeated for each video]

Finally, please answer following questions:

1. **Which video do you remember the most and why?**

______________________________________________________________________________________________________________________________________________________________

1. **In your opinion, what type of information should be shown in a video to reduce suicidal ideation?**

______________________________________________________________________________________________________________________________________________________________

______________________________________________________________________________________________________________________________________________________________

______________________________________________________________________________________________________________________________________________________________

1. **Which video did you choose? Please place every video starting from the 1^st^ place (= best video) to the 4^th^ place (please assign each place only one video, for example please do not place two videos 2^nd^, etc.).**

| ___. Place | Video 1 |
| --- | --- |
| ___. Place | Video 2 |
| ___. Place | Video 3 |
| ___. Place | Video 4 |

[Only three videos were available for rating for the female participants]

__________________________________________________________________________________

Interview Guide Focus Groups

[Main questions are marked in **bold,** the other questions were used as trigger questions if necessary.]

General:

**How did you feel while watching and rating the videos?**

What was good about the videos?

What did you find suboptimal?

**What was important to you while rating these videos? Which criteria were important to you?**

**In your opinion how will the videos affect LGBQ adolescents in their coming out?**

- Self-acceptance
- Sexual identity

To what extent did you find differences in regards to the effects of the videos?

**In your opinion how will the videos affect LGBQ adolescents who face severe adversities during their coming out and may have suicidal ideation?**

Which video did you choose? Which video did you find most suitable?

Ranking of videos:

[Questions referring to the specific video watched]

**How did you feel while watching this video?**

What was good about the video? What did you find suboptimal?

**In your opinion how will the video affect other LGBQ adolescents who are in the course of coming out?**

Structure of suicide prevention videos:

**In your opinion how should videos be structured to reduce suicidal ideation specifically for adolescents in the course of coming out?**

**How could the videos be disseminated?**

Other:

**What would be important to you to tell the group in connection with the topic of the focus group which has not been addressed yet?**

Links of the videos that were discussed in the focus groups

All the videos were produced in German language.

Videos featuring male protagonists (*n=*4):

Video 1: <https://www.youtube.com/watch?v=nxXoHJmqFIU>

Video 2: <https://www.youtube.com/watch?v=DLEkbpSlAFQ&list=PLFi3-q5KBk885f15UFDnF-mai-XZxG4bG&index=9&t=0s>

Video 3: <https://www.youtube.com/watch?v=obc6fk5Xw3Q>

Video 4: <https://www.youtube.com/watch?v=gdKb4PKD_0k>

Videos featuring female protagonists (*n=*3):

Video 1: <https://www.youtube.com/watch?v=n0shGHyczSk>

Video 2: <https://www.youtube.com/watch?v=L-JdQasyM8A>

Video 3: <https://www.youtube.com/watch?v=mDzwsI_SFnk>
